# Retraction: A Case Report of Grisel’s Syndrome Complicating the Postoperative Course of Craniotomy for a Massive Cystic Brain Lesion 

**DOI:** 10.7759/cureus.r157

**Published:** 2025-01-10

**Authors:** Takara R Newsome-Cuby, Kwadwo Darko, Peace Odiase, Sean O'Leary, Charles Marchese, Michael J Valentine, Benjamin Pautler, Usama AlDallal, Mohamed Ismail, Fakhar Hayat

**Affiliations:** 1 School of Osteopathic Medicine, Kansas City University, Kansas City, USA; 2 Neurosurgery, Korle-Bu Teaching Hospital, Accra, GHA; 3 Biochemistry and Cancer Biology, Meherry Medical College, Nashville, USA; 4 Neurosurgery, University of Texas Medical Branch at Galveston, Galveston, USA; 5 School of Medicine, Royal College of Surgeons In Ireland - Medical University of Bahrain, Busaiteen, BHR; 6 Neurosurgery, King Hamad University Hospital, Busaiteen, BHR

This article has been retracted by the Editors-in-Chief due to credible allegations of misconduct in the form of honorary authorship and exclusion of a contributing author. These allegations were investigated and confirmed by the Bahrain Defence Force Royal Medical Services Institutional Review Board. The authors disagree with this retraction.

